# Supplementary material for: CoExpresso: assess the quantitative behavior of protein complexes in human cells
Source: BMC Bioinformatics. 2019 Jan 9;20:17. doi: 10.1186/s12859-018-2573-8 (PMC6327379; doi:10.1186/s12859-018-2573-8)
Supplement: Supplementary file 1 — Supplementary Figures to CoExpresso: Assess the quantitative behavior of protein complexes in human cells. (PDF 5647 kb) [file 12859_2018_2573_MOESM1_ESM.pdf]

Supplementary figures to CoExpresso: Assess the quantitative behavior of protein complexes in human cells

Morteza H Chalabi, Vasileios Tsiamis, Lukas Käll, Fabio Vandin and Veit Schwämmle

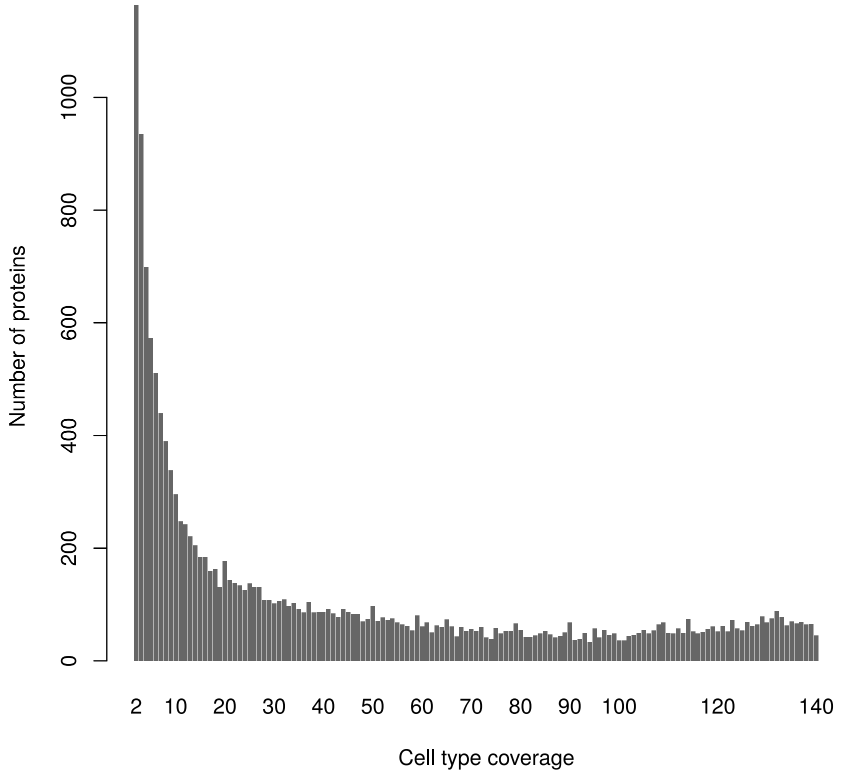

Figure S1: Low coverage of protein profiles across cell types complicates co-expression analysis.

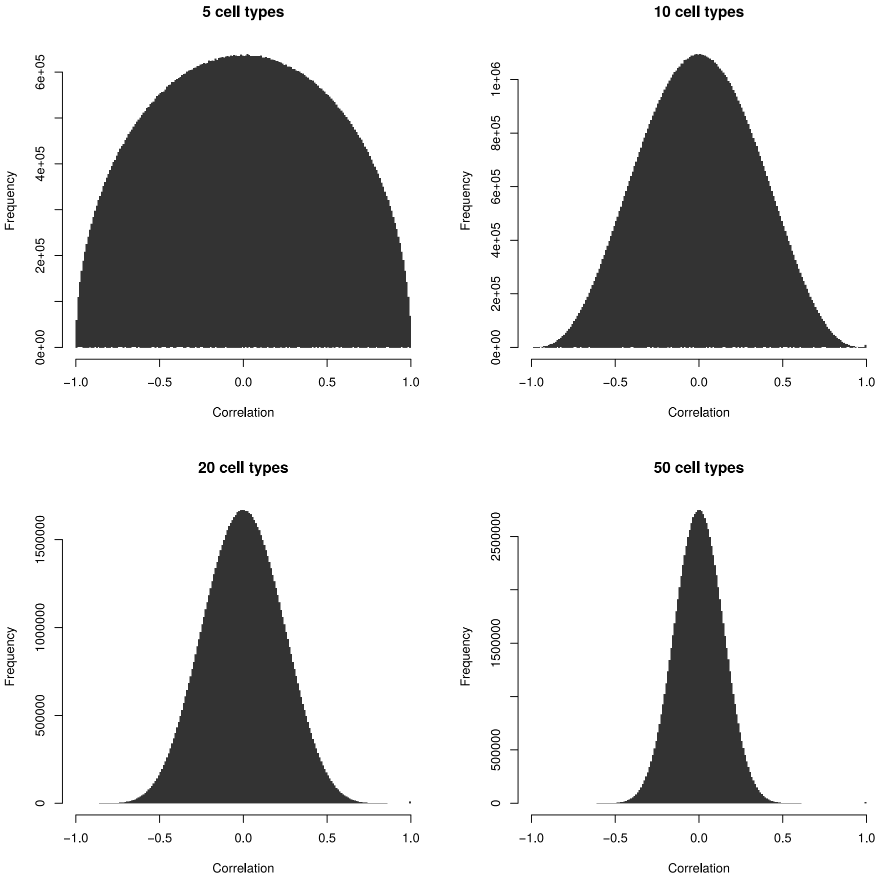

Figure S2: Distribution of Pearson's correlations for normally distributed data. Higher coverage across cell types leads to a more narrow distribution and therefore to higher significance of high correlation values.

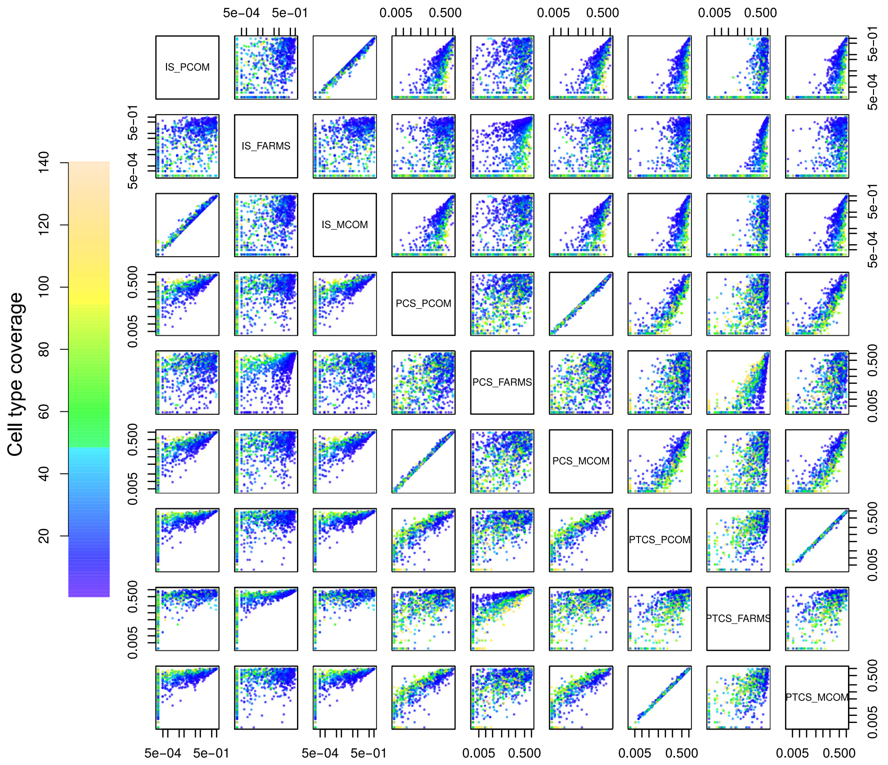

Figure S3: Comparison of complex p-values between models. Colors indicate cell type coverage.

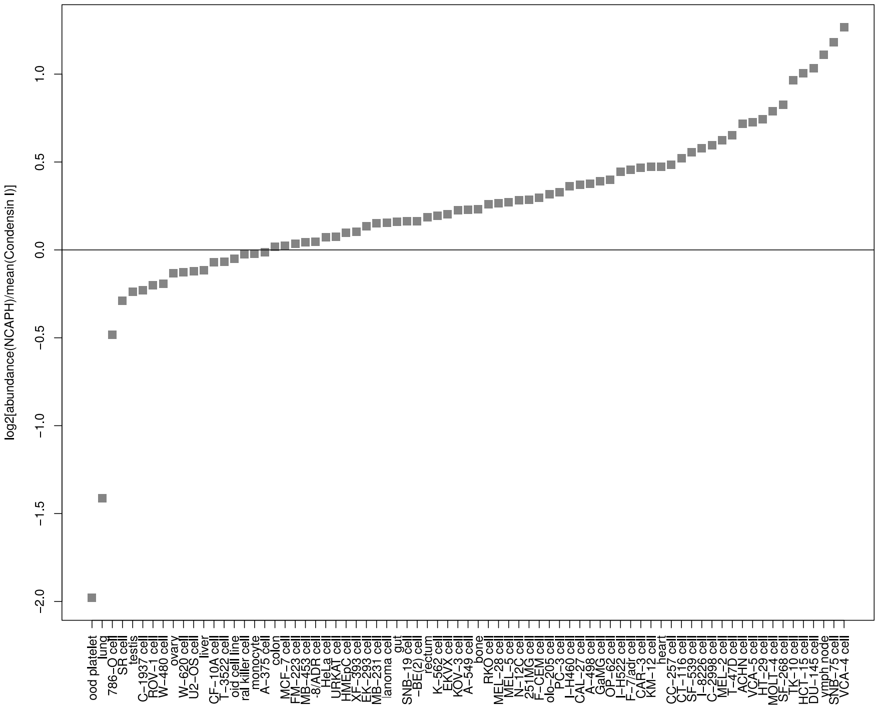

Figure S4: Deviations of NCAPH abundances with respect to common abundance profile of the Condensin I complex, calculated by the mean over all protein subunits.

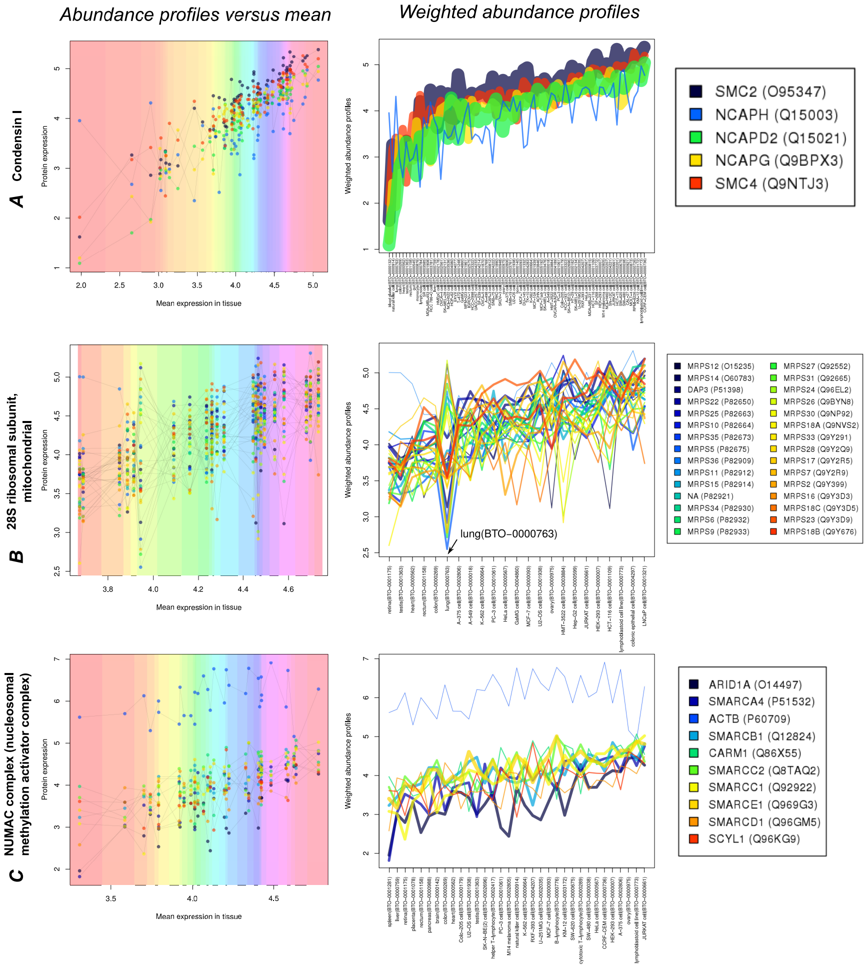

Figure S5: Protein abundance over cell types for 3 protein complexes. Left panels: Abundance is shown versus the mean taken for each cell type (different colors). Middle panels: Protein abundances for the different cell types which were ordered according to the mean expression. Line thickness corresponds to the weights calculated by the FAM model.

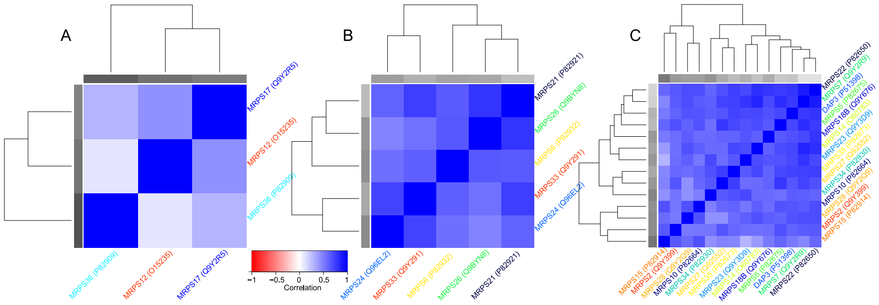

Figure S6: Correlation maps for three different protein subgroups of the S28 mitochondrial ribosomal complex. **A** MRPS17, MRPS36 and MRPS12 show very low correlation when investigated as separate protein group. **B** Group of the five proteins MRPS21, MRPS24, MRPS26, MRPS6 and MRPS33 exhibited highest correlations and reasonably high significance. **C** Correlation map of large protein subgroup comprising DAP3, MRPS2, MRPS5, MRPS7, MRPS10, MRPS14,MRPS15, MRPS18B, MRPS22, MRPS23, MRPS27, MRPS28, MRPS34 and MRPS35.

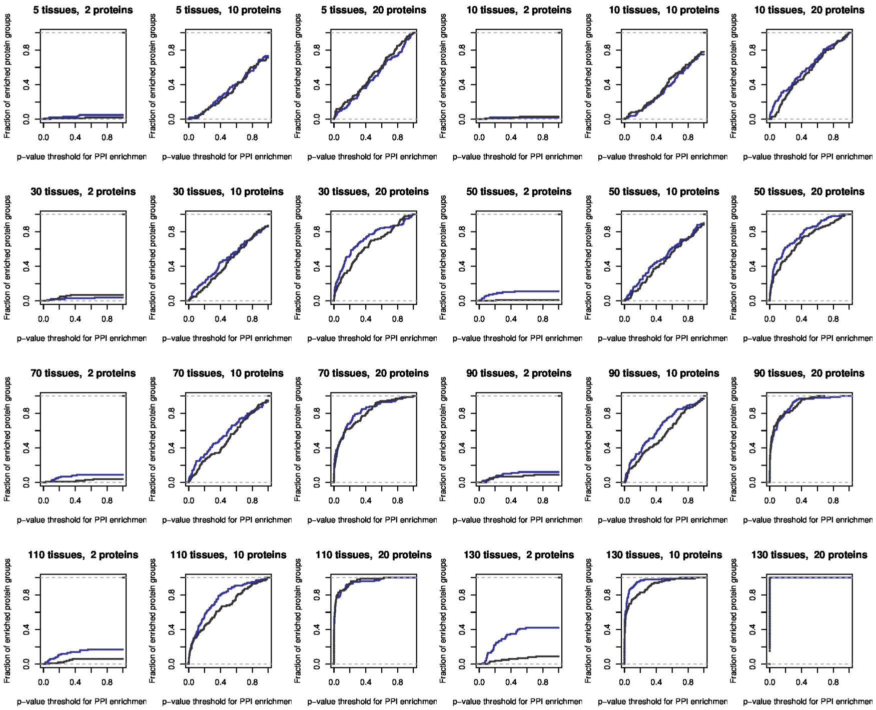

Figure S7: Comparison of network enrichment scores in STRINGdb for best scoring protein groups (blue) versus random protein groups (dark gray). In most cases, high scores lead to recognition of more densely connect proteins, i.e. proteins with known interactions.

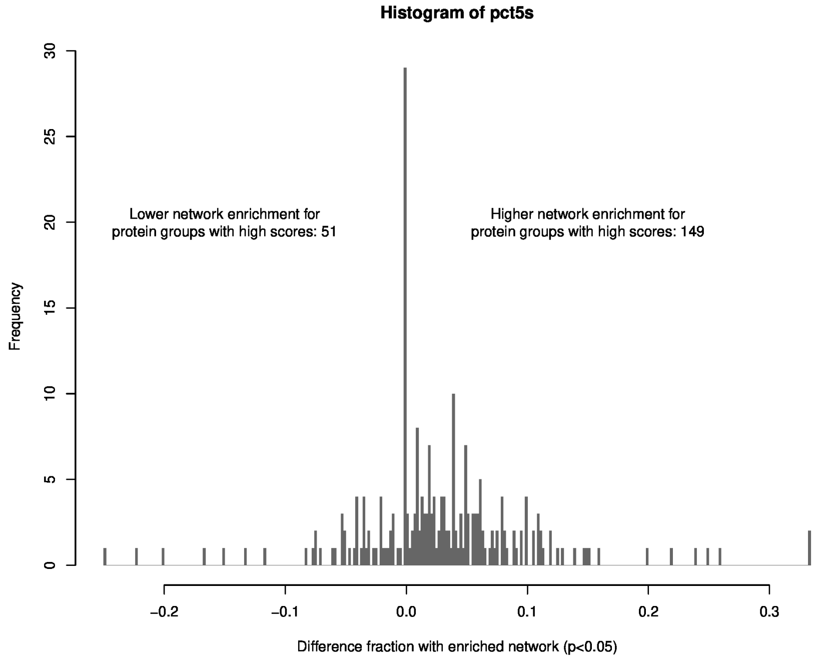

Figure S8: Summary of Fig. S7 by taking the differences between the fraction of best-scoring protein groups with enriched networks (enrichment value $p < 0.05$) and the fraction obtained for randomly chosen protein groups. This was calculated for different cell type coverages and protein numbers. The majority of cases shows higher network enrichment for high scoring protein groups.
